# Visual analysis of image-guided radiation therapy based on bibliometrics: A review

**DOI:** 10.1097/MD.0000000000032989

**Published:** 2023-02-22

**Authors:** Jin-Hui Yuan, Qing-Song Li, Yan Shen

**Affiliations:** a Department of Radiation Oncology, The Second Affiliated Hospital, Zhejiang University School of Medicine, Hangzhou, China.

**Keywords:** bibliometrics, cite space, IGRT, radiotherapy

## Abstract

Radiation therapy plays an important role in tumor treatment. The development of image-guided radiation therapy (IGRT) technology provides a strong guarantee for precise radiation therapy of tumors. However, bibliometric studies on IGRT research have rarely been reported. This study uses literature collected from the Web of Science during 1987 to 2021 as a sample and uses the bibliometric method to reveal the current research status, hotspots, and development trends in IGRT. Based on 6407 papers published from the Web of Science during 1987 to 2021, we utilized Microsoft Excel 2007 and cite space software to perform statistical analysis and visualization of IGRT. A total of 6407 articles were included, this area of IGRT has gone through 4 stages: budding period, growth period, outbreak period, and stationary period. The research category is mainly distributed in Radiology Nuclear Medicine Medical Imaging, which intersects with the research categories of Materials, Physics, and Mathematics. Yin FF, Tanderup K, and Sonke JJ are highly productive scholars who are active in IGRT research, while Jaffray DA, van Herk M and Guckenberger M are authors with high impact in this field. The team of scholars has close cooperation within the team and weak cooperation among teams. The League of European Research Universities, University of Texas System, University of Toronto, and Princess Margaret Cancer are the main research institutions in this field. The United States has the most research literature, followed by China and Germany. Six thousand four hundred seven articles are distributed in 712 journals, and the top 3 journals are *Med Phys, Int J Radiat Oncol*, and *Radiather Oncol*. Precise registration, intelligence, magnetic resonance guidance, and deep learning are current research hotspots. These results demonstrate that the research in this field is relatively mature and fruitful in the past 35 years, providing a solid theoretical basis and practical experience for precision radiotherapy.

## 1. Introduction

The number of new cases and deaths from malignant tumors worldwide is increasing annually, and cancer has evolved into a major public health problem worldwide.^[1]^ As an important means of tumor treatment. It has been reported that 60% to 70 % of cancer patients require different degrees of radiotherapy.^[2]^ Image Guided Radiation therapy (IGRT) is an organic combination of imaging devices and a linear accelerator.^[3]^ Its development declares that radiotherapy has entered the era of precision radiotherapy from traditional radiotherapy. Although the research results in this field increased rapidly in the late 1980s, and literature reviews were reported in various periods,^[4–7]^ the traditional narrative literature review has shortcomings, such as incomplete literature collection and strong subjectivity. With the development of computer science and informatics, bibliometrics provides a new perspective for sorting out the development context and research trends of a certain field while overcoming the shortcomings of narrative reviews. Bibliometrics has certain applications in the field of tumor radiotherapy, mainly focused on heavy ion radiotherapy,^[8]^ intraoperative radiotherapy,^[9]^ lung cancer radiotherapy,^[10]^ head and neck cancer radiotherapy,^[11]^ and radiotherapy and male infertility^[12]^ and other tumors clinical literature metrology research. However, tumor radiotherapy technology-related metrology research remains in the vacuum zone. What is the current status of the IGRT research? What are the research hotspots and challenges? However, these are rarely referenced in the literature. Based on the perspective of bibliometrics, this paper sorts the documents published in the Web of Science Core Collection database from 1987 to 2021 and presents the current research in the IGRT field with a visual map from the aspects of category and publisher distribution, author, institution, country, journal, and document co-citation. The purpose was to provide a research basis and perspective for subsequent related research.

## 2. Materials and methods

### 2.1. Source of data

The study sample data comes from the Web of Science Core Collection and editions including SCI-expanded, SSCI, AHCI, CPCI-S, CPCI-SSH, and E-SCI. Retrieval strategy: TS = “IGRT and Radiation Therapy” OR “IGRT and radiotherapy” OR “Cone beam computed tomography (CBCT) and Radiation Therapy” OR “Image-guided radiation therapy” OR “Image-guided radiotherapy.” Years from 1987 to 2021, and 9570 related records were retrieved.

#### 2.1.1. Quality control.

Three researchers independently searched the literature according to the purpose of the study, and discussed the decision or consulted a third-party expert if there was disagreement.

#### 2.1.2. Elimination criteria.

Cleaning out reviews, conference abstracts, letters, book chapters, and other documents that obviously did not match the study theme.

Finally, 6407 research samples were included, downloading bibliographic information such as authors, institutions, journals, and titles to establish a database on IGRT. The retrieval and download time was April 17, 2022.

### 2.2. Research methods

Bibliometrics is an interdisciplinary science that integrates quantitative knowledge carriers from philology, mathematics, statistics, and other disciplines. It is a method used to explore the structure and regularity of science and technology.^[13]^ The cite space software (https://citespace.podia.com/) was developed by Professor Chaomei Chen of Drexel University in the United States.^[14,15]^ It is a commonly used bibliometric software that intuitively and vividly presents the relationship between documents and is the most important feature. This study used the co-occurrence and co-citation analysis functions of the software (version 5.8. R3 (64-bit)) to visually analyze the research status, related hotspots, and IGRT trends.

Co-occurrence analysis is a type of network cooperation analysis in which authors, institutions, and countries/regions work together to achieve a certain goal in scientific research.^[15,16]^ This method was used to explore the relationship between subjects in the study domain.

Co-citation analysis means that 2 documents are cited by a third document at the same time, and the 2 documents constitute a co-citation relationship.^[8,17]^ Through the co-citation of documents, we can explore the evolution of a particular field.

## 3. Results

### 3.1. Basic distribution characteristics

**Figure 1.**
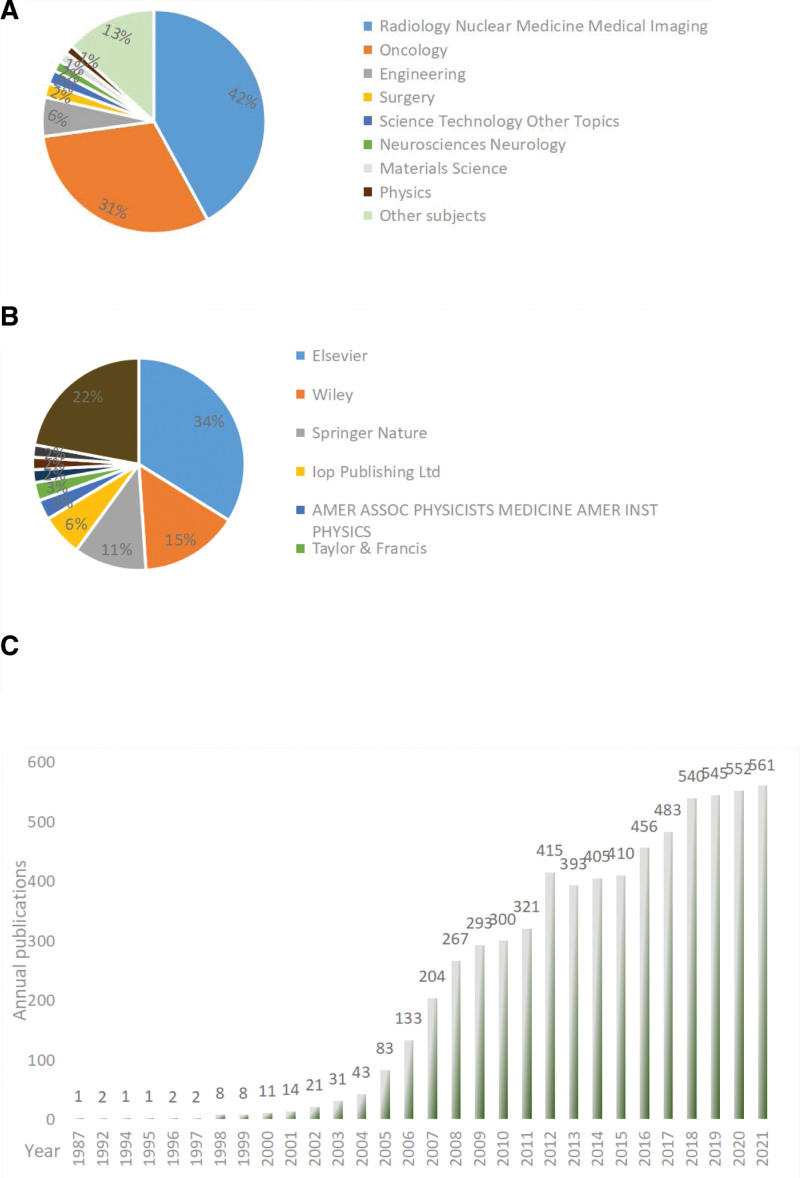
Basic distribution characteristics of IGRT research fields. (A) Distribution characteristics of subject categories; (B) distribution characteristics of publishers; (C) annual distribution of documents. IGRT = image-guided radiation therapy.

The research categories of 6417 articles in this field are mainly distributed in Radiology Nuclear Medicine Medical Imaging, Oncology, and Engineering and intersect with Materials, Physics, and Mathematics; detailed information is provided in Figure 1A.

The research results in this field have been mainly published by 3 publishers: Elsevier, Wiley, and Springer Nature. Among them, Elsevier published about 34 % of the research results on IGRT and is the main publisher in this field, as shown in Figure 1B.

The annual publication of IGRT research literature presents a “J” type (Fig. 1C). It is mainly divided into 4 stages: The germination period (1987–1997), the research in this field has just started, and the number of documents is scarce, basically 1 to 2 documents per year; The growth period (1998–2004), scholars attention increased and research results gradually increased; The outbreak period (2005–2017), the research literature increased rapidly, and the research results were fruitful. It reached a high point in 2012, fell back, and then continued to increase. The stable period (2018–2021), with the research of scholars, shows that the research literature in the field of IGRT is gradually stable and reaches its peak in 2021, with a total of 561 documents. Overall, research results are on the rise.

### 3.2. Cooperation network

**Figure 2.**
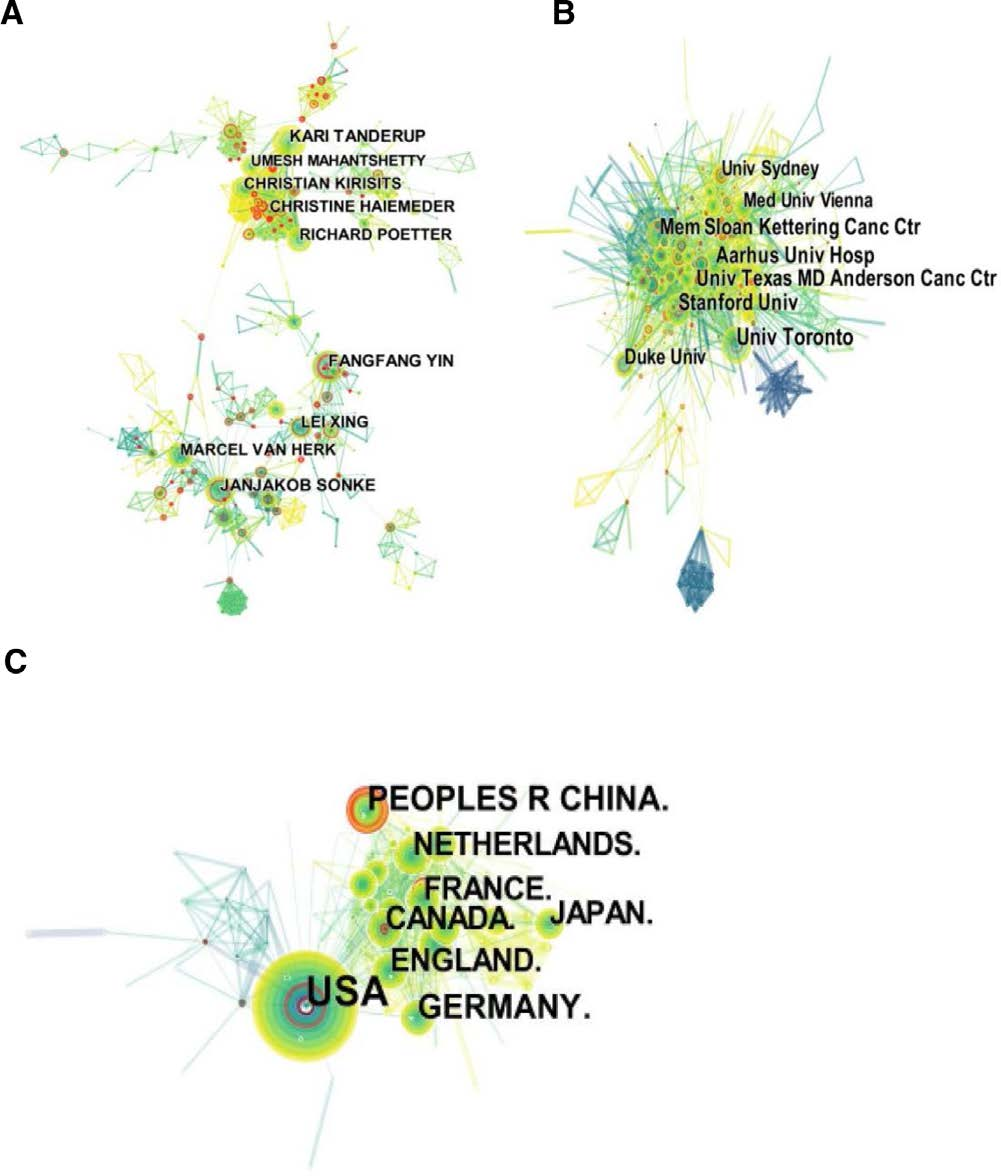
Graph of cooperation network in the field of IGRT (the node size represents the number of documents and the connection line represents the closeness of cooperation). (A) author’s cooperation map; (B) institutional cooperation map; (C) country/region cooperation map. IGRT = image-guided radiation therapy.

The data of 6417 records were imported into cite space software, node select Author, time slice select 1, the network was not cut, and the cooperation network of authors of IGRT was obtained, with 1117 network nodes, 2868 connections, and a density of 0.0046. Scholar Yin FF (n = 65) published the most papers, Tanderup K (n = 63) and Sonke JJ (n = 57) ranked the second and third. Scholars such as Tanderup, Kirisits, and Haiemeder cooperate closely. Sonke JJ and van Herk M cooperate closely and sporadically with the Xing L and Yin FF teams. Scholars such as Poetter R, Xing L, Kirisits C, and Mahatshetty U have published more than 40 papers, and the above scholars are also high-yield scholars who are active in the field, as shown in Figure 2A and Table 1.

**Table 1:** Top 10 authors in terms of volume and citation frequency.

No.	Author	Publications	Cited author	Citations
1	Yin FF	65	Jaffray DA	771
2	Tanderup K	63	van Herk M	754
3	Sonke JJ	57	Guckenberger M	500
4	Poetter R	54	Zelefsky MJ	485
5	Xing L	50	Keall PJ	452
6	van Herk M	48	Murphy MJ	417
7	Kirisits C	47	Langen KM	413
8	Haiemeder C	42	Kupelian PA	397
9	Mahantshetty U	40	Sonke JJ	387
10	Jaffray DA	35	Yan D	358

Similarly, node chooses an institution to obtain an institutional cooperation network map (Fig. 2B). From the map, it can be seen that institutions in this field cooperate closely and form a certain academic scale. The League of European Research Universities is the primary research institution. Institutions such as the University of Texas System, the University of Toronto, and the Princess Margaret Cancer Centre have published more than 200 papers and are important institutions in the field of IGRT, as shown in Table 2.

**Table 2:** Top 10 countries and institutions on IGRT.

Country/region	Publications	Institution	Publications
USA	2526	League of European Research Universities	518
Peoples R China	629	University of Texas System	333
Germany	601	University of California System	267
Canada	531	University of Toronto	264
Netherlands	495	Unicancer	246
England	402	University Health Network Toronto	208
Japan	385	Utmd Anderson Cancer Center	205
France	368	Princess Margaret Cancer Centre	204
Italy	317	Aarhus University	189
Australia	298	Harvard University	182

IGRT = image-guided radiation therapy.

Figure 2C shows the cooperation network map of the country/region on IGRT. The USA had the largest number of papers (n = 2526) (Table 2), and the research results were plentiful. There is extensive cooperative research with Peoples R China, Germany, England, and other countries, but the cooperation is loose and not close enough.

### 3.3. Co-citation network

**Figure 3.**
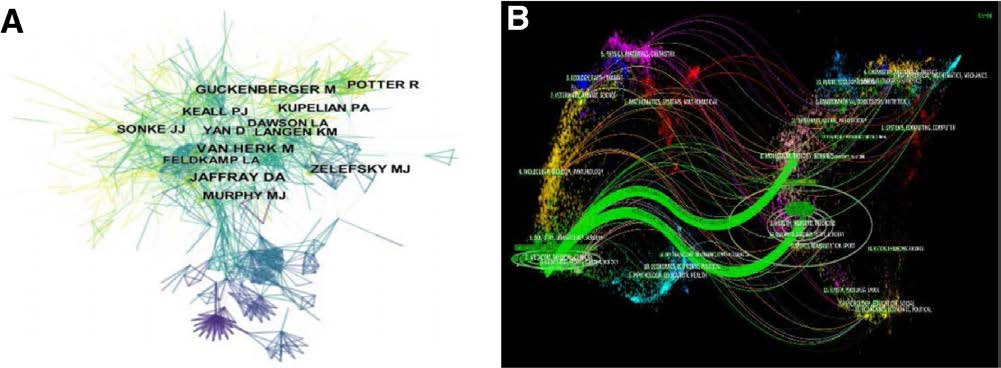
Co-citation network map of IGRT (The node size represents the citation frequency, and the line represents the citation intensity). (A) Co-cited map of authors; (B) dual-map overlay of IGRT. IGRT = image-guided radiation therapy.

The data of 6417 studies were imported into the cite space software, node select Cited Author, time slice select 1, and Pathfinder for the network. A co-citation network of authors was obtained, with 1332 network nodes, 4481 connections, and a density of 0.0051 (Fig. 3A). The papers published by Jaffray DA (n = 771) had the highest citation frequency, followed by van Herk M and Guckenberger M, as shown in Table 1. Scholars van Herk M, Feldkamp LA, Jaffray DA team, Guckenberger M, Keall PJ team, and Potter R team are highly cited teams in this field, and their research results have laid the theoretical foundation and clinical practice of IGRT.

The 6417 sample articles were published in 712 journals. Figure 3B shows a dual-map overlay of the journals in this field. The left side of the map represents the journal distribution of citing documents, which is mainly distributed in medical-clinical, molecular, biology, veterinary, science, physics, and other subjects. The right side of the map represents the journal distribution of the cited literature. Research in this field mainly refers to Molecular - Biology - Genetics, Health – Nursing - Medicine; Chemistry, Mathematical, Systems, Psychology, and other subjects, which also constitute the research basis of this field. *Med Phys, Int J Radiat Oncol*, and *Radiother Oncol* ranked among the top 3 in terms of the number of papers published (Table 3). This is the main reason for the results in this field. *Int J Radiat Oncol*, an authoritative journal in the field of radiotherapy, has the highest citation frequency, followed by *Radiother Oncol, Med Phys*.

**Table 3:** Top 10 journal publications and citations.

No.	Journal	Publications	Cited journal	Citations
1	*Med Phys*	723	*Int J Radiat Oncol*	5532
2	*Int J Radiat Oncol*	656	*Radiother Oncol*	4387
3	*Radiother Oncol*	441	*Med Phys*	3769
4	*Phys Med Biol*	366	*Phys Med Biol*	3031
5	*J Appl Clin Med Phys*	315	*J Clin Oncol*	1864
6	*Radiat Oncol*	218	*Radiat Oncol*	1706
7	*Strahlenther Onkol*	140	*Acta Oncol*	1639
8	*Acta Oncol*	138	*Semin Radiat Oncol*	1616
9	*Brit J Radiol*	123	*Brit J Radiol*	1396
10	*Phys Medica*	111	*J Appl Clin Med Phys*	1328

### 3.4. Literature co-citation analysis

**Figure 4.**
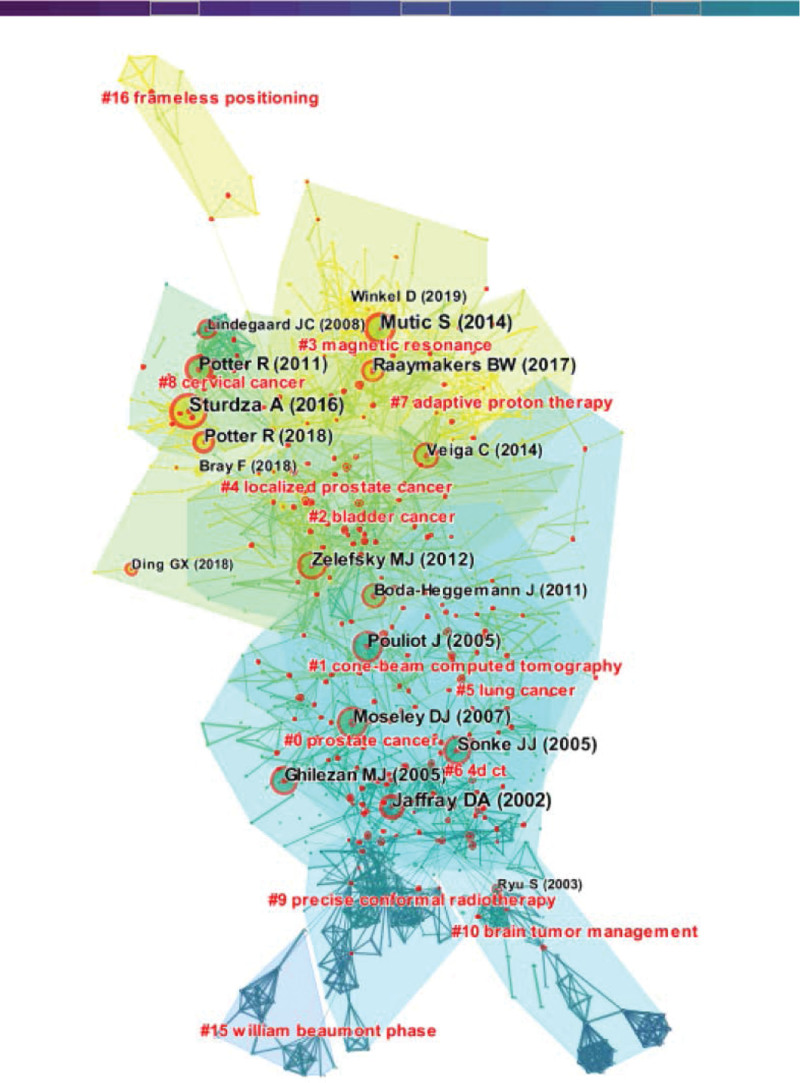
Literature co-citation network map and clustering information.

There were 86,113 references in 6417 articles, with an average of 13.41 references per article. The literature records were imported into the cite space software, node select Reference, time slice select 1, and the co-citation network map of the reference was obtained, with 1756 network nodes, 8329 connections, and a density of 0.0054. The modularity Q value was 0.785, the Weighted Mean Silhouette S value was 0.9088, the clustering structure was significant, and homogeneity was high. Using the LLR clustering algorithm,16 main clusters were obtained (Fig. 4). #0, as the largest cluster, focuses on topics such as “deformable image registration, scatter correction, CBCT, deep learning”; the 3 clusters with dense and significant clustering structures are #8, #9, and #10. #8 cluster mainly focuses on topics such as “Image-guided radiotherapy, therapy, computed tomography, lung, stereotactic radiosurgery”; #9 cluster mainly focused on topics such as “quality assurance, magnetic resonance-guided radiotherapy, multileaf collimator, obi, quality control”; #10 cluster focuses on topics such as “High dose rate brachytherapy, radiobiology, non-small cell lung cancer, 4-dimensional dose calculation, biological outcomes.” The latest cluster is #16, with men year of 2017, with topics focused on “frameless positioning, precise positioning, surface-guided radiation therapy, breath-hold patient positioning”; followed by #3, mean year 2016, research topics focused on “MR-guided radiotherapy, low-field mr-linac, magnetic field.”

### 3.5. Betweenness centrality

Centrality is an indicator of the importance of hub nodes connecting 2 fields. The higher the value, the more important the literature.^[15]^ Table 4 shows the 10 articles with the highest betweenness centrality on IGRT. The literature with the highest betweenness centrality comes from Potter (2011) in Cluster 8 and Liang (2019) in Cluster 7. Among the 10 articles, 5 articles are from cluster 7, and 3 articles are from cluster 0.

**Table 4:** References with the highest centrality.

Rank	Centrality	References	Cluster ID
1	0.45	Potter R, 2011, *Radiother Oncol*, 100, 116^[18]^	8
2	0.45	Liang X, 2019, *Phys Med Biol*, 64, 0^[19]^	7
3	0.44	Veiga C, 2016, *Int J Radiat Oncol*, 95, 549^[20]^	7
4	0.42	Kurz C, 2016, *Med Phys*, 43, 5635	7
5	0.41	Ghilezan MJ, 2005, *Int J Radiat Oncol*, 62, 406	0
6	0.41	Hansen DC, 2018, *Med Phys*, 45, 4916	7
7	0.38	Kuban DA, 2008, *Int J Radiat Oncol*, 70, 67	0
8	0.38	Veiga C, 2014, *Med Phys*, 41, 0	7
9	0.38	Nijkamp J, 2008, *Int J Radiat Oncol*, 70, 75	0
10	0.37	Acharya S, 2016, *Int J Radiat Oncol*, 94, 394	3

### 3.6. Citation bursts

Table 5 shows 10 papers with high burst value in this field. Among them, 4 papers are from cluster 1, and its theme focuses on “megavoltage cone-beam CT, cervical cancer, kilovoltage cone-beam CT”; there are 3 articles in cluster 8 and 2 articles in cluster 3. The highest bursts value is the literature published by the scholar Jaffray DA, in which the structure principle and physical properties of the CBCT system of Elekta Oncology Systems are described in detail; the second is the application of IGRT in the radiation therapy of cervical cancer published by the scholar Sturdza A, bursts value is 47.63.

**Table 5:** References with the strongest bursts.

Rank	Bursts	References	Cluster ID
1	56.34	Jaffray DA, 2002, *Int J Radiat Oncol*, 53, 1337^[21]^	1
2	47.63	Sturdza A, 2016, *Radiother Oncol*, 120, 428^[22]^	8
3	43.8	Mutic S, 2014, *Semin Radiat Oncol*, 24, 196	3
4	43.2	Potter R, 2011, *Radiother Oncol*, 100, 116	8
5	31.08	Zelefsky MJ, 2012, *Int J Radiat Oncol*, 84, 125	4
6	30.58	Mackie TR, 2003, *Int J Radiat Oncol*, 56, 89	1
7	29.92	Lindegaard JC, 2013, *Acta Oncol*, 52, 1510	8
8	29.19	Raaymakers BW, 2017, *Phys Med Biol*, 62, 0	3
9	28.53	Pouliot J, 2005, *Int J Radiat Oncol*, 61, 552	1
10	27.37	Islam MK, 2006, *Med Phys*, 33, 1573	1

### 3.7. Most cited references

The most cited literature is an indicator to measure the influence of literature, which usually refers to literature with high academic value and professional influence.^[13,15]^ Table 6 lists the top 10 most cited papers in this field of IGRT; these articles were published from 2000 to 2011, mainly from 2005 to 2007. The most cited paper was published in Med Phys in 2008, with 1211 citations; Followed by Benedict, SH (2010) published in Med Phys, with 1046 citations. The research topics of the highly cited literature focus on 3 levels; The structure, principle, and development of IGRT, The practical application of IGRT in the radiotherapy of tumors in various parts, and; The new technology of tumor radiotherapy. Among them, Shirato (2000) elaborated on the construction principle, geometric performance, and clinical application of IGRT; Wang (2005) improved the registration accuracy by optimizing the image registration algorithm to ensure the effect of radiotherapy; Jaffray and DA (2002) further elaborated the physical properties of the image guidance system by taking the image acquisition system of Elekta Oncology Systems as an example, and they laid a theoretical foundation for the clinical application of IGRT. Maier-Hauff (2007) and Gerszten (2007) used IGRT for clinical research on spinal metastases and glioma, respectively, fully affirmed the status of IGRT technology in precise radiotherapy and laid a practical foundation for the subsequent extensive development of this technology, which is widely used in the central nervous system and soft tissue imaging because of its excellent soft tissue resolution and functional imaging. The fusion of magnetic resonance (MR) systems and linear accelerators has become a popular research topic in recent years. For example, Poetter (2011) achieved gratifying clinical benefits of MR-guided treatment in cervical cancer patients.

**Table 6:** The top 10 references by citation counts on IGRT.

No.	Title	First author	Journal	Year	Citations
1^[23]^	Volumetric modulated arc therapy: IMRT in a single gantry arc	Otto, K	*Med Phys*	2008	1211
2^[24]^	Stereotactic body radiation therapy: The report of AAPM Task Group101	Benedict,S H.	*Med Phys*	2010	1046
3^[21]^	Flat-panel cone-beam computed tomography for image-guided radiation therapy	Jaffray,DA	*Int J Radiat Oncol*	2002	984
4^[18]^	Clinical outcome of protocol based image (MRI) guided adaptive brachytherapy combined with 3D conformal radiotherapy with or without chemotherapy in patients with locally advanced cervical cancer	Poetter,R	*Radiother Oncol*	2011	547
5^[25]^	Respiratory correlated cone beam CT	Sonke,JJ	*Med Phys*	2005	509
6^[26]^	Intracranial thermotherapy using magnetic nanoparticles combined with external beam radiotherapy: Results of a feasibility study on patients with glioblastoma multiforme	Maier-Hauff,K	*J Neuro-Oncol*	2007	492
7^[27]^	Radiosurgery for spinal metastases - Clinical experience in 500 cases from a single institution	Gerszten,PC	*Spine*	2007	482
8^[28]^	Physical aspects of a real-time tumor-tracking system for gated radiotherapy	Shirato,H	*Int J Radiat Oncol*	2000	472
9^[29]^	Validation of an accelerated “demons” algorithm for deformable image registration in radiation therapy	Wang,H	*Phys Med Biol*	2005	464
10^[30]^	Cone-beam computed tomography with a flat-panel imager: magnitude and effects of x-ray scatter	Siewerdsen,JH	*Med Phys*	2001	460

IGRT = image-guided radiation therapy, MRI = magnetic resonance imaging.

### 3.8. Theme evolution in radiotherapy

**Figure 5.**
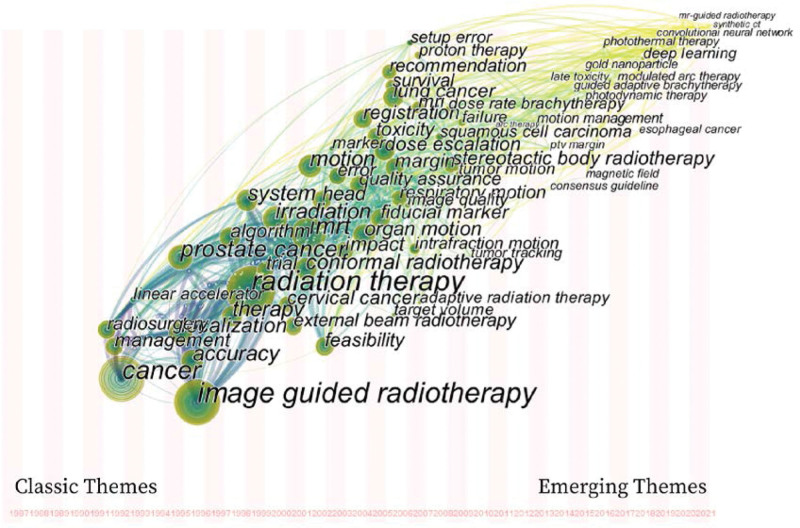
Theme evolution map of IGRT. IGRT = image-guided radiation therapy.

The time zone map in cite space software can vividly and intuitively show the evolution of research hotspots and core topics in a field over time.^[14,15]^ The high-frequency keywords in the IGRT research were imported into the software to generate the evolution trend map (Fig. 5). Figure 5 shows topics such as cancer, management, radiosurgery, image-guided radiotherapy, prostate cancer, tomotherapy, linear accelerators, and accuracy from 1991 to 2000. Among these, image-guided surgery, comformal radiation therapy, radiation therapy, and other studies that appeared in 1996 to 2000 have evolved into classic themes in this field. With the attention and in-depth research of scholars, organ motion, quality assurance, margin, toxicity, small volume, dose escalation, stereotactic radiotherapy, recommendation, magnetic resonance imaging, dose rate, image quality, arc therapy, etc, have become hot topics in 2001 to 2016. Since 2017, modulated arc therapy, deep learning, synthetic CT, MR-guided radiotherapy, nanoparticles, and convolutional neural networks have become current research hotspots and emerging topics in this field.

## 4. Discussion

According to the basic distribution characteristics, the research direction in this field mainly focuses on Radiology Nuclear Medicine Medical Imaging, and Oncology, which intersect with Computer Science and other directions. This is consistent with the essence of IGRT technology, that is, the development of IGRT technology is closely related to the development of medical imaging and computer technologies. Currently, AI is rapidly developing, and this technology is still being actively explored in the field of IGRT. At present, it focuses on target delineation and motion management ^[31,32]^ and further breakthroughs and cross-studies are needed in the future. Overall, the number of documents in this field has increased linearly, indicating that scholars continue to pay attention to the development of this field and that the research results are fruitful. In the process of research and development, it has experienced the general rules of scientific research, such as germination period, growth period, outbreak period, and stable period; for example, research in this field has just started. Dempsey PK (1992) affirmed the application status of IGRT technology in the treatment of pineal tumors.^[33]^ With the increasing attention of scholars, research topics are more diversified, and the research literature is increasing rapidly and gradually reaching its peak. After the peak, scholars’ topics became more rational and focused more on scientific issues, and the number of documents declined.

From the perspective of cooperation networks, scholars have a certain academic scale in this field, and cooperation within the team of scholars is close. Yin FF, Xing L, Sonke JJ, van Herk M, Poetter R, Kirisits C, Haiemeder C, and others are high-yield scholars in the field of IGRT research and are relatively active, which promotes the development and application of IGRT technology. However, the cooperation between the teams is not close enough and is scattered, and cooperation between the teams needs to be strengthened in the future, especially in multi-center cooperation research. The League of European Research Universities is a leading institution in this field. Soochow University, Washington University, Ludwig Maximilians University, Munchen, University Manchester, and Chinese Acad Sci have become the hot research institutions on IGRT in recent years. USA has the largest number of publications and has the greatest contribution to IGRT research; the research results mainly focus on the basic theory, equipment research and development, and clinical application of IGRT technology. In recent years, national scientists such as Peoples R China and India have been active in this field, contributing to the clinical application and technological development of IGRT in cancer treatment in their own countries.

From the perspective of co-cited networks, an author’s co-cited network is dense and frequently cited. The papers published by Jaffray DA have the highest citation frequency, while van Herk M and Guckenberger M rank second and third, respectively. They are high-impact authors in this field, laying a theoretical foundation and accumulating rich practical experience for the development of IGRT technology. Research literature was distributed across 712 journals. The *International J of Radiotherapy Oncol*, a professional authoritative journal in the field of tumor radiotherapy, is cited much more frequently than other journals. *Radiother Oncol* and *Med Phys* are the second and third most frequently cited journals, respectively, and are the main carriers of high-quality research results in this field. The research hotspots reflected by the highly cited literature in this field mainly include; The technical principle and physical properties of IGRT; The clinical application of IGRT technology in tumor radiotherapy; The integration, development, and application of IGRT technology and other technologies; and combining centrality and burst value. Potter R (2011),^[18]^ Liang X (2019)^[19]^ and Sturdza A (2016)^[22]^ are the frontier articles in this field, and their research results have pushed the development of IGRT technology to a new height.

From the perspective of topic evolution, the hot topics in this field have successively been the application of classical topics such as “radiation therapy, cancer, radiosurgery, and image-guided radiotherapy (IGRT) in head cancer and cervical cancer.” Gradual evolution to emerging hot topics such as “Stereotactic body radiotherapy, gold nanoparticles, deep learning, photothermal therapy, MR-guided radiotherapy, and synthetic CT”. There are many themes in a certain period on the evolution map, indicating that there are many research results, reflecting that the field is in a period of prosperity; on the contrary, in the trough. There were few research topics before 1999, mainly focusing on “radiotherapy” and “clinical application of radiosurgery,” which belonged to the enlightenment stage of research in this field. From 2000 to 2012, research topics were diverse and intensive. Among these, 2000 was a key year in the field of IGRT research. Phillips, MH (2000)^[34]^ verified the repeatability and accuracy of the device by combining an image-guided positioning device with a linear accelerator, opening a precedent for modern integrated IGRT equipment. Gilberg PL (2000) ^[35]^ and Martinez AA (2000) ^[36]^ studied the application of IGRT in brain tumors and prostate cancer, laying a clinical foundation for the clinical application of IGRT in other tumors. Shirato, H (2000) ^[28]^ and Jaffray DA (2000) ^[37]^ described the physical properties of the IGRT technology in detail. After 2013, scholars gradually became more rational in this field, with more scientific topics and fewer research topics. These characteristics coincided with the 4 periods of annual IGRT distribution and conformed to the general rules of scientific research. Emerging themes have expanded the extension and depth of classic themes, such as research on the application of deep learning in positioning and image improvement.^[38–40]^ The application of new technologies or their combinations confirms inheritance and innovation among the topics in this field. For example, the combination of optical surface monitoring system and CBCT can improve the positioning accuracy of head, neck, and chest tumors, and reduce additional radiation.^[41,42]^

In summary, this paper reviews the literature on IGRT research from 1987 to 2021 and analyzes the overall status, research hotspots, and trends of IGRT research from the perspective of category, annual distribution, journal distribution, and author cooperation network for the first time. IGRT technology is currently widely used in tumor radiotherapy and has made great contributions to tumor precise radiotherapy. In the future, the fusion of new technologies with IGRT and bioinformatics-guided radiotherapy may become a hot research topic and direction in this field. There are some shortcomings in this study: First, data duplication, although the research data has been de-duplicated by software and manual, it is unavoidable to miss it; Second, the database was single, and some databases such as PubMed, Embase, and Medline were not analyzed, since there were many repetitions between these databases and the articles in the WOS database, it might not have a significant impact on the final results; Third: cite space software itself defects, for statistical data may be biased. Subsequently, other databases are combined to improve the mining and analysis of IGRT research hotspots and trends.

## Author contributions

**Supervision:** Jin-Hui Yuan.

**Visualization:** Jin-Hui Yuan.

**Writing – original draft:** Jin-Hui Yuan, Qing-Song Li, Yan Shen.

**Writing – review & editing:** Jin-Hui Yuan, Qing-Song Li.
